# Predictive value of gadoxetic acid–enhanced MRI for posthepatectomy liver failure: a systematic review

**DOI:** 10.1007/s00330-021-08297-8

**Published:** 2021-09-25

**Authors:** Qiang Wang, Anrong Wang, Ernesto Sparrelid, Jiaxing Zhang, Ying Zhao, Kuansheng Ma, Torkel B. Brismar

**Affiliations:** 1 Division of Medical Imaging and Technology, Department of Clinical Science, Intervention and Technology (CLINTEC), Karolinska Institutet, Stockholm, Sweden; 2 Department of Radiology, Karolinska University Hospital Huddinge, 141 86 Stockholm, Sweden; 3 Department of Hepatobiliary Surgery, People’s Hospital of Dianjiang County, Chongqing, China; 4 Division of Surgery, Department of Clinical Science, Intervention and Technology (CLINTEC), Karolinska Institutet, Karolinska University Hospital, Stockholm, Sweden; 5 Department of Pharmacy, Guizhou Provincial People’s Hospital, Guiyang, Guizhou Province China; 6 Experimental Cancer Medicine, Clinical Research Center, Karolinska Institutet, Stockholm, Sweden; 7 Clinical Research Center (KFC) and Center for Allogeneic Stem Cell Transplantation (CAST), Karolinska University Hospital Huddinge, Stockholm, Sweden; 8 Institute of Hepatobiliary Surgery, Southwest Hospital, Army Medical University, Chongqing, China

**Keywords:** Magnetic resonance imaging, Liver failure, Gadolinium ethoxybenzyl DTPA, Hepatectomy, Systematic review

## Abstract

**Objectives:**

Effective and non-invasive biomarkers to predict and avoid posthepatectomy liver failure (PHLF) are urgently needed. This systematic review aims to evaluate the efficacy of gadoxetic acid–enhanced MRI-derived parameters as an imaging biomarker in preoperative prediction of PHLF.

**Methods:**

A systematic literature search was performed in the databases of PubMed/Medline, Web of Science, Embase, and Cochrane Library up to 11 December 2020. Studies evaluating the incidence of PHLF on patients who underwent hepatectomy with preoperative liver function assessment using gadoxetic acid–enhanced MRI were included. Data was extracted using pre-designed tables. The Quality In Prognostic Studies (QUIPS) tool was adopted to evaluate the risk of bias.

**Results:**

A total of 15 studies were identified for qualitative synthesis and most studies were marked as low to moderate risk of bias in each domain of QUIPS. The most commonly used parameter was relative liver enhancement or its related parameters. The reported incidence of PHLF ranged from 3.9 to 40%. The predictive sensitivity and specificity of gadoxetic acid–enhanced MRI parameters varied from 75 to 100% and from 54 to 93% in ten reported studies. A majority of the studies revealed that the gadoxetic acid–enhanced MRI parameter was a predictor for PHLF.

**Conclusions:**

Gadoxetic acid–enhanced MRI showed a high predictive capacity for PHLF and represents a promising imaging biomarker in prediction of PHLF. Multicenter, prospective trials with large sample size and reliable, unified liver function parameters are required to validate the efficacy of individual liver function parameters.

**Key Points:**

• *There is an obvious heterogeneity of the published studies, not only in variance of MRI liver function parameters but also in indication and extent of the liver resection.*

• *Signal intensity (SI)–based parameters derived from gadoxetic acid–enhanced MRI are the commonly used method for PHLF prediction.*

• *Gadoxetic acid–enhanced MRI-derived parameters showed high predictive efficacy for PHLF and can potentially serve as a predictor for the incidence of PHLF.*

**Supplementary Information:**

The online version contains supplementary material available at 10.1007/s00330-021-08297-8.

## Introduction

Posthepatectomy liver failure (PHLF) remains the leading cause of perioperative morbidity and mortality [1]. The reported incidence depends on different PHLF criteria and individual medical center’s experience but is typically about 10% [2], although as high frequency as 43% has been reported [3].

In order to evaluate liver function, there are several commonly used tests or scoring models, such as the blood biochemical tests, the indocyanine green (ICG) retention test, the LiMAx® test, Child–Pugh score, and the Model for End-stage Liver Disease (MELD) score. However, those only give information on certain specific aspects of liver function or on global liver function [4]. Their value and application in the prediction of PHLF are hampered by the fact that they do not consider the heterogeneous distribution of liver function among different liver segments, which is significant in patients with fibrosis/cirrhosis or after chemotherapy [5]. Although regional liver function information can be provided by hepatobiliary scintigraphy, its wide utilization is mainly limited by its low spatial resolution [6].

As an important predictor for PHLF, the future liver remnant (FLR) volume is most often calculated using CT-derived volumetry [7]. However, simple volumetry cannot take liver function into account so the thresholds for achieving a safe margin are roughly adjusted for prior chemotherapy, hepatosteatosis, and cirrhosis [5]. In a normal liver, a lower limit of the FLR volume as low as 20% can be accepted, while in settings of steatosis or post-chemotherapy, the FLR should be 30–35%, and in case of liver cirrhosis, at least 40% of FLR is generally considered to be required [8]. When the FLR is lower than those thresholds, several techniques such as portal vein ligation or portal vein embolization are used to cause a reactive growth response before extensive liver resection [4].

Gadoxetic acid (Primovist®, Bayer Healthcare)–enhanced MRI is used as a routine preoperative workup for liver lesion detection and characterization [9]. After venous injection, as much as 50% of this contrast medium is exclusively taken up by organic anion-transporting polypeptides (OATPs) on sinusoidal membrane of normal hepatocytes and then excreted into the biliary ducts [10]. Liver parenchymal enhancement is determined by the equilibrium of this transport mechanism with an optimal enhancing effect observed during a period of 10–40 min after administration [11].

Previous studies have shown that with the progression of liver disease the expression of OATPs decreases, making it suitable for quantitative evaluation of liver function [10, 12]. Theoretically, gadoxetic acid–enhanced MRI has a potential advantage in the prediction of PHLF as it can provide both volumetric and functional information of the FLR [13, 14]. A number of studies on PHLF prediction using gadoxetic acid–enhanced MRI have been conducted, but their reported efficacy for PHLF incidence varies. However, there has not been any systematic summary about the imaging findings and the prognostic value of gadoxetic acid–enhanced MRI for PHLF. Such summaries are of importance for the evidence-based management of patients. The present systematic review aims to summarize the approaches used for the prediction of PHLF by gadoxetic acid-enhanced MRI, describing their prognostic performance and providing a picture of the current landscape of research in the prediction of PHLF. In addition, as most studies compared the gadoxetic acid-enhanced MRI parameters and ICG test in the prediction of PHLF, their overall efficacy has also been summarized in this systematic review.

## Materials and methods

This systematic review was registered on the PROSPERO website (https://www.crd.york.ac.uk/prospero/, registration no. CRD42020200602) and performed in accordance with the Preferred Reporting Items for Systematic Reviews and Meta-Analyses (PRISMA) guidelines (Supplement 1) [15].

### Literature search

A systematic literature search was conducted in databases of PubMed/Medline, Embase, Web of Science, and the Cochrane Library from inception until 11 December 2020 to identify eligible studies. Terms used in literature retrieval were: liver failure/dysfunction/insufficiency, hepatectomy, and gadoxetic acid–enhanced MRI (Supplement 2). To also incorporate potential literature, cited references in the included studies were manually examined.

All studies satisfying the following criteria were included: (1) observational study (retrospective or prospective); (2) patients who underwent partial hepatectomy of at least one Couinaud segment; (3) liver function parameters quantitatively assessed by gadoxetic acid–enhanced MRI used as a predictor for PHLF, solely or as a parameter in a model; (4) clearly stated definition of PHLF; (5) published in English.

The exclusion criteria were as follows: (1) articles in the forms of review, reference abstracts, letters, editorials, and case reports; (2) animal studies; (3) gadoxetic acid–enhanced MRI only used for liver volumetry; (4) other treatments, such as chemotherapy, transarterial chemoembolization, or portal vein embolization (PVE) between MRI exam and hepatectomy.

### Study selection and data extraction

The decision to include or exclude a publication was made by reading its title and abstract according to the prespecified criteria. The excluded studies should meet at least one item of the exclusion criteria or were totally irrelevant. To avoid the removal of potentially relevant literature, the full text was obtained to further evaluate its eligibility (Supplement 3).

The following data of each included study were extracted: (1) study characteristics including first author, publication year, regions; (2) patient characteristics such as the number of patients, age, indication for hepatectomy, extent of hepatectomy, PHLF criteria, and cases of PHLF; (3) MRI characteristics, gadoxetic acid–enhanced MRI derived parameters, and their corresponding formula; (4) predictors and its cutoff value, predictive accuracy data; (5) ICG test results. When several liver functional parameters were evaluated concomitantly in one study, the optimal one was selected. For duplicate data based on the same study subjects from one institution, the most informative publication was included. To reduce the high variability in terminology and to facilitate readability, we normalized the terms describing the same concept but expressed in various forms among different publications.

### Risk of bias assessment

The risk of bias among the included studies was assessed using the Quality In Prognosis Studies (QUIPS) tool [16]. The bias was evaluated in the domains of study participation, study attrition (waived in this review as no follow-up information was required), measurement of prognostic factor, outcome measurement, research confounding, and statistical analysis and reporting. The results of every domain were ranked as high, moderate, and low risk.

The literature search, study selection, data extraction, and literature quality assessment were performed independently and cross-validated by two reviewers to control the potential bias. When disagreement occurred, it was solved by a discussion under the supervision of a senior researcher.

### Criteria of PHLF

The widely used definition of PHLF, proposed by the International Study Group of Liver Surgery (ISGLS), defines PHLF as an increased international normalized ratio (> 1.2) and hyperbilirubinemia (> 22 μmol/L or above preoperative value) on postoperative day 5 or afterwards [17]. Another commonly used definition of PHLF is the “50–50” criteria [18]. It defines PHLF as when prothrombin time is < 50% and serum bilirubin is > 50 μmol/L on postoperative day 5 or later. A less common criterion defines PHLF as hepatic encephalopathy with hyperbilirubinemia (total bilirubin > 4.1 mg/dL), international normalized ratio > 2.5, and ascites with drainage volume > 500 mL/day [19].

## Results

### Study characteristics

**Fig. 1:**
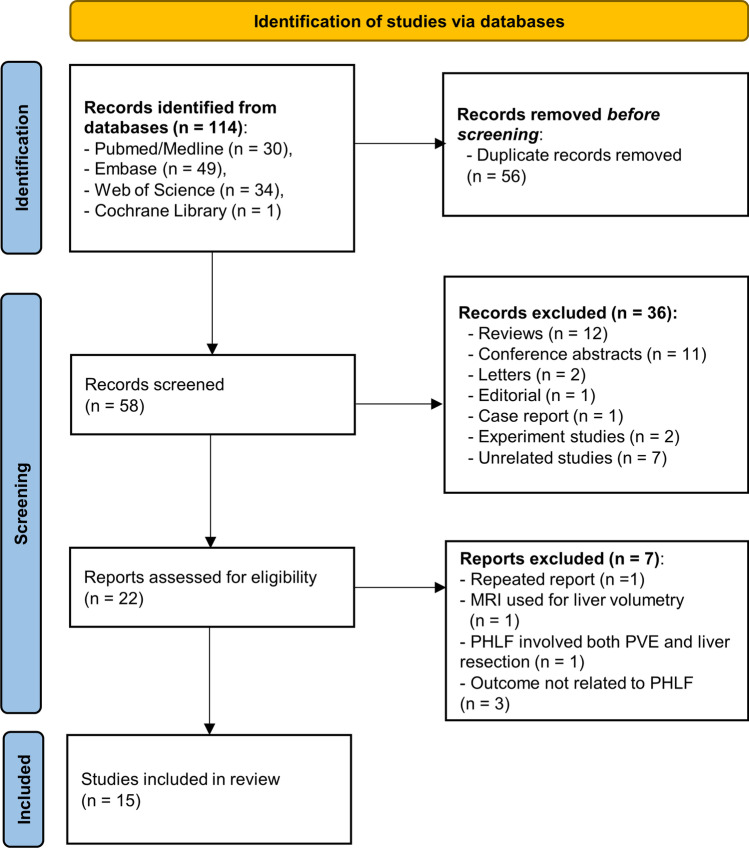
Flow chart of the process of study selection

Out of a total of 114 studies found in the systematic literature search, 15 studies using gadoxetic acid–enhanced MRI-derived parameters to predict PHLF were considered eligible (Fig. 1). The 15 included studies were published between August 2011 and September 2020. The study sample size ranged from 11 to 192 patients and comprised in total of 1327 patients. Except for one prospective study, all studies were retrospective. All studies were conducted in single centers. Table 1 illustrates the study characteristics of included studies [20–33, 35].

**Table 1:** Study and patient characteristics

Study ID	Year	Region	Study design	Consecutive	Number of Patients	Age (median/mean)	Chronic liver disease (%)	PVE before MRI exam	Main indications‡	Extent of liver resection	PHLF Criteria	Cases of PHLF (%)
Cho[20]	2011	Korea	R	Unclear	29	57	48% (cirrhosis 21%)	NA	HCC (48%)	Major§	ISGLS	7 (24%)
Wibmer[21]	2013	Austria	R	Unclear	73	64.4	NA	NA	CRLM (71%)	Major	“50–50”; ISGLS	3 (4%); 29 (40%)
Sato[22]	2015	Japan	R	Unclear	11†	59.5	NA	Yes	Liver metastases (52%)	Major	ISGLS	7 (waived)
Jin[23]	2016	Korea	R	Unclear	121	56	92% (cirrhosis 61%)	NA	HCC (100%)	Minor§ + major	“50–50”; ISGLS	7 (5.8%); 38 (31%)
Costa[24]	2017	Canada	R	Yes	65	60.8	NA	NA	CRLM (71%)	Major	ISGLS	9 (14%)
Asenbaum[25]	2018	Austria	R	Unclear	62	59.8	NA	NA	CRLM (53%)	Major	ISGLS	16 (26%)
Chuang[26]	2018	Taiwan	R	Yes	115	60	100%	No	HCC (78%)	Minor + major	ISGLS	16 (14%)
Kim[27]	2018	Korea	R	Yes	73	59.7	100% (cirrhosis 47%)	NA	HCC (100%)	Minor + major	ISGLS	18 (25%)
Theilig[28]	2019	Germany	P	Yes	36	62	NA	Yes	CRLM (31%);HC (31%)	Major	ISGLS	14 (39%)
Araki[29]	2020	Japan	R	Unclear	129 (SCoh) + 26 (VCoh)	67 (SCoh)	NA	Yes	Liver metastases (43%)	Minor + major	ISGLS	9 (6%)^#^
Donadon[30]	2020	Italy	R	Yes	137	65	18%	Yes	CRLM (77%)	Minor + major	ISGLS	22 (16%)
Orimo[31]	2020	Japan	R	Unclear	140 (SCoh) + 52 (VCoh)	65 (SCoh); 72 (VCoh)	NA	NA	HCC (69%)	Major (≥ 2 sections)	ISGLS	49 (26%)
Zhu[32]	2020	China	R	Unclear	101	55 (men), 53 (women)	100% (cirrhosis 23%)	NA	HCC (100%)	Major	Defined by encephalopathy etc ¶	15 (15%)
Tsujita[33]	2020	Japan	R	Unclear	41	66	66%	NA	HCC (100%)	Major + minor	ISGLS	16 (39%)
Wang[34]	2020	China	R	Unclear	116	49.0	86% (cirrhosis 62%)	No	HCC (100%)	Major + minor	ISGLS	28 (22%)

† the number of patients for a second analysis; ‡ the most frequent indication with its percentage is listed while the exclusive indication is marked as 100%; § “major liver resection” refers to three or more Couinaud segments, while “minor liver resection” to less than three Couinaud segments; ¶ posthepatectomy liver failure is defined as encephalopathy, with hyperbilirubinemia (total bilirubin > 4.1 mg/dL), international normalized ratio > 2.5, and ascites with drainage volume > 500 mL/d; # refers to the incidence of PHLF grade B, C, in which clinical management after operation is altered while it remains the same in grade A of PHLF; *CRLM*, colorectal liver metastases; *HC*, hilar cholangiocarcinoma; *HCC*, hepatocellular carcinoma; *ISGLS*, the International Study Group of Liver Surgery criteria; *NA*, not available; *P*, prospective study; *PHLF*, posthepatectomy liver failure; *PVE*, portal vein embolization; *R*, retrospective study; *SCoh*, study cohort; *VCoh*, validation cohort

### Risk of bias assessment

**Fig. 2:**
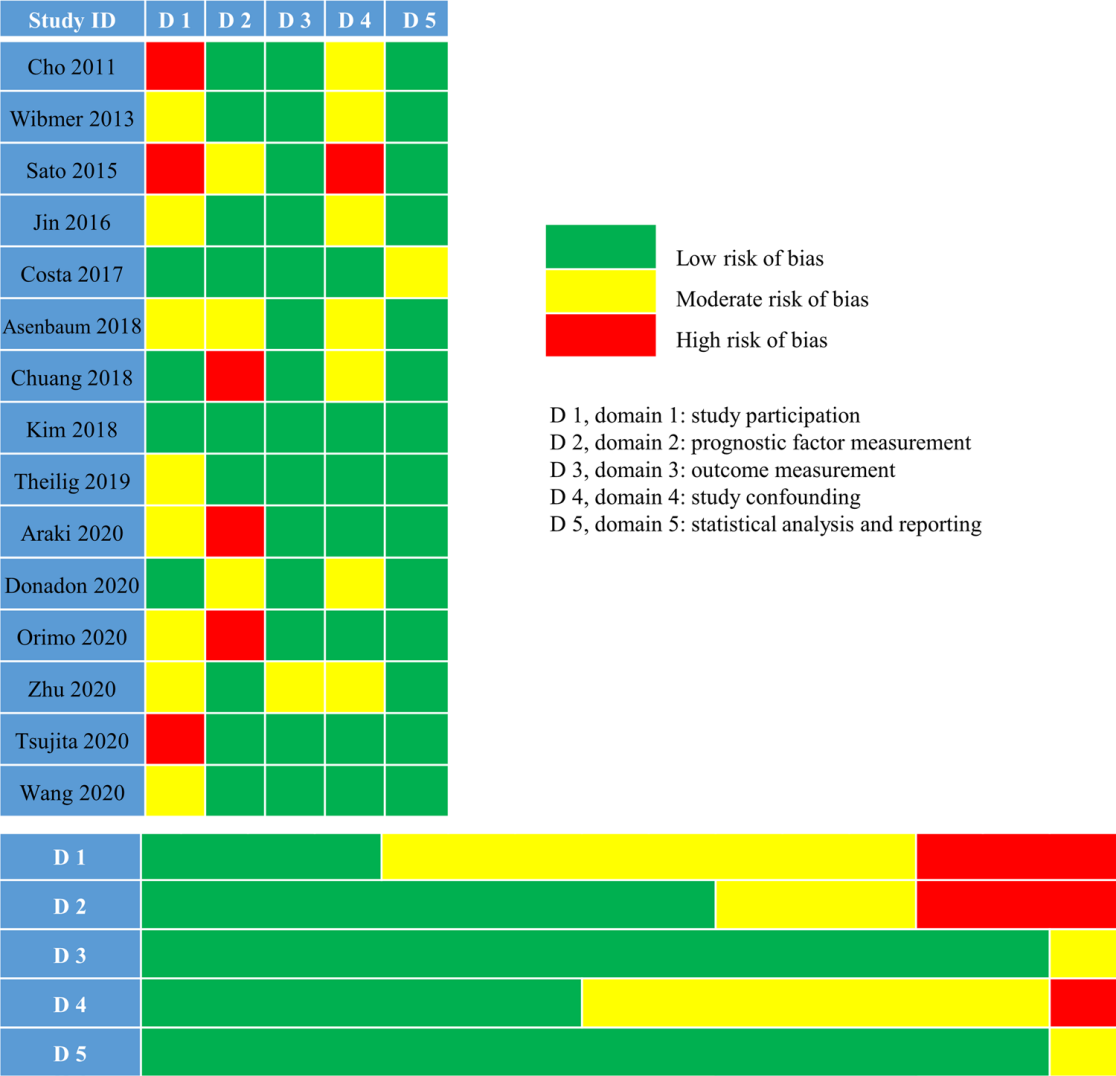
Risk of bias assessment using the QUIPS tool (the study attrition domain waived as no follow-up information was required)

In general, most studies showed a low to moderate risk of bias in each domain. Briefly, in the study participation domain, three studies were marked as high risk as they had a limited number of participants (< 50 cases) coming from a single center and also did not state whether the patients were enrolled consecutively or not [20, 22, 33]. In the domain of prognostic factor measurement, three studies were labeled as high risk of bias as they did not demonstrate the interval between MRI exam and hepatectomy, the number of reviewers, and whether blinded to the clinical outcome or not [26, 29, 31]. Regarding the outcome measurement domain, one study showed moderate risk as it applied a less-common criterion for PHLF in which a subjective index, encephalopathy, was included [32]. In the term of study confounding, half of the studies showed a moderate or high risk of bias as they did not measure all important confounders, among them three studies also did not perform multivariate analysis [20, 22, 30]. In the domain of statistical analysis and reporting, one study was marked as moderate risk as it did not state the *p* value during univariate and multivariate regression analysis [24]. The summary of the risk of bias evaluated by the QUIPS tool is demonstrated in Fig. 2.

### Patient characteristics

The average age of the participants in the included 15 studies was between 49 and 71.5 years with predominantly male patients in twelve studies (12/15). Four studies (4/15) evaluated the incidence of PHLF in patients with pretreatment of PVE. Five studies (5/15) exclusively assessed PHLF after HCC resection, and one of them focused on HCC with portal vein invasion [33]. Two studies focused on PHLF after resection of primary liver diseases; the incidence of PHLF after “mixed indications” was assessed in the remaining eight studies. Eight papers evaluated PHLF after major liver resection (three or more Couinaud segments), while the remaining seven studies evaluated PHLF after both minor (less than three Couinaud segments) and major liver resections. Detailed patient characteristics are provided in Table 1.

### Incidence of PHLF

The ISGLS criteria of PHLF were applied in 14 studies, while a less common definition of PHLF was adopted in one study [32]. Among the 14 studies, two of them further compared the incidence of PHLF defined by “50–50” criteria [21, 23]. As one study evaluated PHLF after PVE as a second analysis and consisted of only 11 patients, whereof 7 cases experienced PHLF [22], the incidence was waived since we considered that it could not represent the real situation. The incidence of PHLF among the other 14 studies ranged from 3.9 to 40% by ISGLS criteria and were 4 to 5.8% respectively in the two by “50–50” criteria (Table 1).

### MRI characteristics

Nine studies performed MRI using a 3.0-T scanner, five studies used a 1.5-T, whereas one study used both [24]. The dose of gadoxetic acid was in accordance with the clinical dosage in all studies (0.025 mmol/kg or 0.1 mL/kg). All but two studies [26] obtained the hepatobiliary phase at 20 min after contrast medium injection. The MRI characteristics of the studies included are summarized in Supplementary Table 1.

### Gadoxetic acid–enhanced MRI-derived liver function parameters

Relative liver enhancement (RLE) or RLE-related parameters were the most frequently used gadoxetic acid–enhanced MRI-derived parameters with seven studies evaluating them (7/15). Seven papers assessed the efficacy of a compound parameter combining liver volume and gadoxetic acid–enhanced MRI-based liver function in the prediction of PHLF. Hepatic uptake index (HUI), a gadoxetic acid–enhanced MRI-derived parameter also taking liver volume into account, was evaluated in four studies (4/15). Five studies quantified liver function of the whole liver, eight studies of the FLR, one study measured both, while the remaining one evaluated it of the FLR and of the resected part of the liver separately. One study (1/15) adopted radiomics approach and another (1/15) used dynamic hepatocyte-specific contrast-enhanced MRI (DHCE-MRI) (Table 2).

**Table 2:** Characteristics of gadoxetic acid–enhanced MRI-derived parameters

Study ID	Quantitative parameter	Quantified liver region	Formula	Interval between MRI and hepatectomy	No. of readers	Blindness to clinical data
Cho 2011	RLE and remRLE	Whole and FLR	(SI_HBP_—SIpre)/SIpre	< 4 weeks	2	Yes
Wibmer 2013	RLE(%)	Whole	(SI_HBP_—SIpre)/SIpre * 100	≤ 8 weeks	1	Yes
Sato 2015	remRE Index	FLR	(SI_HBP_—SIpre)/SIpre * 100 * remLV	Mean of 20 days	2	Unclear
Jin 2016	RLE(%)	Whole	(SI_HBP_—SIpre)/SIpre * 100	≤ 4 weeks	1	Yes
Costa 2017	RLE	Whole	(SI_HBP_—SIpre)/SIpre	≤ 16 weeks	1	Yes
Asenbaum 2018	functFLR	FLR	(FLR(%)*remRLE)/body weight	≤ 10 weeks	1	Yes
Chuang 2018	remCER	FLR	(SI_HBP_—SIpre)/(SI_TP_—SIpre)	Unclear	Unclear	Unclear
Kim 2018	rHUI-BW	FLR	remLV * ((remSI_L20_ / SI_S20_) − 1)/body weight * 1 000	≤ 4 weeks	1	Yes
Theilig 2019	remRLE	FLR	(SI_HBP_—SIpre)/SIpre	14 and 4 days before hepatectomy	2	Yes
Araki 2020	FRLV(LMR)	FLR	((remSI_L20_ / remSI_Lpre_)/(SI_M20_ / SI_Mpre_)*remLV) /BSA	Unclear	Unclear	Unclear
Donadon 2020	HUI	Whole	LV*(SI_L20_ / SI_S20_—1)	Unclear	Unclear	Yes
Orimo 2020	rHUI-BSA	FLR	remLV * ((remSI_L20_ / SI_S20_) − 1)/BSA	Unclear	Unclear	Unclear
Zhu 2020	Radiomics	Whole	NA	≤ 1 week	2	Yes
Tsujita 2020	rHUI and HUI	FLR and resected liver	rHUI = remLV * ((remSI_L20_ / SI_S20_) − 1); HUI = rHUI + ((TFLV-remLV)* ((resSI_L20_ / SI_S20_) − 1))	≤ 8 weeks	1	Yes
Wang 2020	RF_UR_, sRF_UR_	FLR	NA (based on dynamic contrast enhanced MRI)	≤ 1 month	2	Unclear

*BSA*, body surface area; *FLR*, future liver remnant; *FRLV(LMR)*, functional remnant liver volume corrected by liver-muscle ratio; *functFLR*, functional FLR; *HUI*, hepatic uptake index; *LV*, liver volume; *NA*, not available; *remCER*, contrast enhancement ratio of the liver remnant; *remLV*, the remnant liver volume; *remRE Index*, relative enhancement index of the liver remnant; *remRLE*, relative liver enhancement of the liver remnant; *remSI*_*Lpre*_, signal intensity of the liver remnant before contrast medium injection; *remSI*_*L20*_, signal intensity of the liver remnant at 20 min after contrast medium injection; *resSI*_*L20*_, signal intensity of the resected liver at 20 min after contrast medium injection; *rHUI*, hepatic uptake index of the liver remnant; *rHUI-BSA*, rHUI corrected by BSA; *rHUI-BW*, rHUI corrected by body weight; *RF*_*UR*_, sum of the uptake rate of the remnant liver regions; *RLE*, relative liver enhancement; *SI*_*HBP*,_ liver signal intensity in hepatobiliary phase; *SI*_*L20*_, signal intensity of the liver at 20 min after contrast medium injection; *SI*_*M20*_, signal intensity of the muscle at 20 min after contrast medium injection; *SI*_*Mpre*_, signal intensity of the muscle before contrast medium injection; *SI*_*pre*_, liver signal intensity before contrast medium injection; *SI*_*S20*_, signal intensity of the spleen at 20 min after contrast medium injection; *SI*_*TP*_, liver signal intensity in transitional phase; *sRF*_*UR*_, sum of the uptake rate of the remnant liver standardized by standard liver volume; *TFLV*, total functional liver volume

The reported interval between the MRI exam and the liver resection varied from 4 days to 16 weeks, but it was not mentioned in four studies. In eleven papers at least one experienced radiologist was among the readers. In five studies, it was unclear whether readers were blinded to the clinical data or not during image analysis (Table 2).

### Predictive performance of PHLF

Thirteen studies (13/15) provided the predictive accuracy of various gadoxetic acid–enhanced MRI parameters for PHLF, with AUC (area under the receiver operating characteristic curve) ranging from 0.67 to 0.96 and accuracy from 0.80 to 0.88 in four reported studies. The sensitivity and specificity were calculated in eleven studies (11/15), varying from 75 to 100% and from 54 to 93%, respectively. Eleven studies (11/15) supplied optimal cutoff values, among which one study verified it using a separate validation cohort with an accuracy of 0.80 [29]; another study yielded a sensitivity of 89% and a specificity of 92% in the validation cohort [31]. Detailed information about predictive accuracy of PHLF evaluated by gadoxetic acid–enhanced MRI parameters is listed in Table 3.

**Table 3:** Predictive accuracy of gadoxetic acid–enhanced MRI parameters

**Study ID**	Predictor(s)	Cut-off value	Sensitivity (%)	Specificity (%)	AUC (95% CI)	Accuracy (95% CI)
Cho 2011	RLE + remRLE	RLE: 0.84 remRLE: 0.73	85.7	RLE: 77.3 remRLE: 81.8	0.84	0.84 (0.65–0.95)
Wibmer 2013	RLE(%)	NA	NA	NA	NA	NA
Jin 2016	RLE(%)	82.36	100	54.4	0.79 (0.65–0.92)	NA
Costa 2017	RLE	NA	NA	NA	0.665	0.875
Sato 2015	remRE Index	NA	NA	NA	NA	NA
Chuang 2018	remCER	1.23	87.5	62.6	0.78 (0.69–0.85)	NA
Theilig 2019	remRLE	-0.044	75	92.6	0.854	NA
Araki 2020	FRLV(LMR)	615	100 (VCoh)	77.3(VCoh)	SCoh: 0.939† (0.891–0.987)	0.808(VCoh)
Asenbaum 2018	functFLR	8.73	94	76	0.904 (0.803–0.977)	NA
Kim 2018	rHUI-BW	12.38	94.4	90.9	0.956 (0.877–0.991)	NA
Donadon 2020	HUI	574.33	98	83	0.84 (0.71–0.92)	NA
Orimo 2020	rHUI-BSA	0.313	75 (VCoh)	78.12(VCoh)	SCoh: 0.80	NA
Tsujita 2020	rHUI and rHUI/HUI	NA	NA	NA	0.962 (0.908–1) †	NA
Zhu 2020	Radiomics	0.712	93.3	77.9	0.894 (0.823–0.964)	0.802 (0.713–0.869)
Wang 2020	sRF_UR_	0.0176	77.3	92.9	0.882 (0.809–0.934)	NA

† Grade B, C PHLF versus no/grade A PHLF, in which clinical management after operation is altered in grade B, C PHLF while it remains the same in grade A of PHLF; *AUC*, area under the receiver operating characteristic curve; *FLR*, future liver remnant; *FRLV(LMR)*, functional remnant liver volume corrected by liver-muscle ratio; *functFLR*, functional FLR; *HUI*, hepatic uptake index; *PHLF*, posthepatectomy liver failure; *remCER*, contrast enhancement ratio of liver remnant; *remRE Index*, relative enhancement index of liver remnant; *remRLE*, RLE of the liver remnant; *rHUI*, HUI of the liver remnant; *rHUI-BSA*, rHUI corrected by body surface area; *rHUI-BW*, rHUI corrected by body weight; *RLE*, relative liver enhancement; *NA*, not available; *SCoh*, study cohort; *sRF*_*UR*_, sum of the uptake rate of the remnant liver standardized by standard liver volume; *VCoh*, validation cohort

### ICG test between PHLF and non-PHLF groups

An additional comparison was performed to evaluate the performance of ICG relevant tests. A majority of the included studies (11/15) compared the results of ICG test or its related parameters between PHLF and non-PHLF groups. Only five of those showed a significant difference: ICG-R15 test in two studies [32], ICG-plasma disappearance rate (ICG-PDR) test and its related parameters in one study [27], ICG clearance–related parameter in one study^22^, and both ICG-R15 and ICG-PDR tests in one study [33]. No studies showed a significantly greater AUC of ICG-test than that of gadoxetic acid–enhanced MRI parameters. The two studies that evaluated compound parameters integrating the ICG test and FLR volume together, creating ICG-Krem (ICG clearance of the FLR)- [22] or ICG-PDR-related parameters (ICG-PDR*FLR and (ICG-PDR*FLR)/body weight) [27] showed that those could discriminate PHLF from non-PHLF significantly (Table 4).

**Table 4:** Results of ICG-R15 test between PHLF and non-PHLF groups

**Study ID**	PHLF		Non-PHLF		*p* value	
	Cases	% (mean or median)	Cases	% (mean or median)		AUC
Cho 2011	7	12.32 ± 5.25	22	13.07 ± 5.15	Not sig	0.54
Sato 2015	7	ICG-Krem: 0.07	4	ICG-Krem:0.101	Sig	NA
Jin 2016	7	11.5 (1.9–22.0)	114	7.0 (0.0–68.8)	Not sig	NA
Asenbaum 2018	16	4.25 (3.55–7.0) ICG-PDR: 21.2 (17.8–24.7)	46	2.75 (1.0–6.0) ICG-PDR: 25.0 (18.0–30.3)	Not sig	0.35 (ICG-PDR: 0.65)
Chuang 2018	16	10.4	99	7.6	Not sig	NA
Kim 2018	18	ICG-PDR: 0.12 ± 0.04 ICG-PDR*FLR: 64.19 ± 37.67 ICGPDR*FLR/BW: 0.95 ± 0.51	55	ICG-PDR:0.16 ± 0.03 ICG-PDR*FLR: 137.81 ± 69.39 ICG-PDR*FLR/BW: 2.14 ± 1.06	Sig	0.75 (ICG-PDR)
Araki 2020	5†	SCoh: 9.1 (8.1–12.8) †	124†	SCoh: 11.2 (1.8–42.0) †	Not sig	0.57
Orimo 2020	29	SCoh: 12.1 (2.3–87.8)	111	SCoh: 10.7 (2.6–94.2)	Not sig	NA
Zhu 2020	15	8.2 (1.3–28.4)	86	12.8 (6.1–18.0)	Sig	NA
Tsujita 2020	9†	15.7 (12.8–20.9)†; ICG-PDR: 10.5 (9.6–12.9)†	32†	11.1(6.6–15.3) †; ICG-PDR:14.2 (12.1–16.5) †	Sig	0.78 (ICG-PDR: 0.76)
Wang 2020	28	6.5 (3.8–9.8)	88	3.1 (2.1–5.3)	Sig	0.77

† Grade B, C PHLF versus no/grade A PHLF, in which clinical management after operation is altered in grade B, C PHLF while it remains the same in grade A of PHLF; *AUC*, area under the curve; *ICG-Krem*, indocyanine green clearance of the liver remnant; *ICG-PDR*, indocyanine green plasma disappearance rate; ICG-PDR*FLR, ICG-PDR multiply by the volume of future liver remnant (FLR), and (ICG-PDR*FLR)/BW is ICG-PDR*FLR corrected by body weight; *ICG-R15 test*, ICG retention rate at 15 min after injection; *PHLF*, posthepatectomy liver failure; *NA*, not available; *SCoh*, study cohort; *Sig*, significant with *p* < 0.05

## Discussion

The current systematic review reveals that quantitative liver function parameters derived from gadoxetic acid–enhanced MRI exhibit encouraging efficacy in the prediction of PHLF, although the efficacy requires verification from future prospective, large samples studies using standardized parameters.

The approach employed in the included studies for preoperative liver function assessment is based on the measurement of liver parenchyma enhancement, in which three approaches can be achieved: signal intensity (SI) of liver parenchyma, MR relaxometry, and DHCE-MRI [6, 10]. SI can be obtained directly using region of interest measurements, while MR relaxometry requires additional imaging sequences and DHCE-MRI involves both additional imaging sequences and complicated data analysis. As it is much less complicated to use, simple SI measurements are widely used in research. In fact, most of the gadoxetic acid–enhanced MRI-derived parameters evaluated in the 15 studies reported in this review belong to SI-related parameters. However, the drawbacks of the SI method are that it is a relative parameter on an arbitrary scale and it can be influenced by many technical factors for example the type of MRI scanner [13]. To overcome that limitation current research focuses on the MR relaxometry for the assessment of the liver function, as it represents a reliable and objective parameter, being independent of MR equipment used after adjustment for magnetic field strength. However, there has not yet been any study evaluating the efficacy of MR relaxometry-related parameters in the prediction of PHLF. More complex techniques such as radiomics and DHCE-MRI can also be used to evaluate liver function and predict the possibility of PHLF, but as these techniques involve complex modeling and powerful computation, they will be more difficult to implement in clinical routine.

Gadoxetic acid–enhanced MRI-derived parameters applied in the included studies vary. The parameters were proposed based on a different rationale. Firstly, to correct for MRI scanner settings and coil, SI-related parameters are usually corrected by internal tissue standards, such as vertebral muscle or spleen [10]. Due to its simplicity, RLE, calculated from the SI of liver parenchyma before and 20 min after contrast medium administration, is a commonly used parameter [10]. Secondly, the measured volumes of the liver parenchyma for SI measurement often vary: some studies focused on the residual part left after liver resection (i.e., the FLR), while others assessed the whole liver. Thirdly, as the FLR volume is the main factor predicting PHLF [20], it seems reasonable to postulate that the compound parameters combining FLR volume and MRI liver function parameter should be superior to the MRI liver function parameter alone. Half of the included studies evaluated parameters involving liver volume for the prediction of PHLF. HUI is an example of such parameters, being determined by liver volume and SI of liver and spleen [35]. Lastly, further correction of MRI liver function parameters can also be made by standardization of body weight or body surface area. In summary, various liver function parameters have been used in the different publications, which creates difficulties in comparisons among them and makes meta-analysis currently impossible. Future research comparing the efficacy of approaches of SI and MR relaxometry, as well as DHCE-MRI and radiomics in the evaluation of liver function and prediction of PHLF, is required.

An extensive body of research has confirmed the correlation between gadoxetic acid–enhanced MRI parameters and the ICG test [13, 14]. However, the use of the ICG test for the prediction of PHLF has been controversial [21, 36, 37] as it just supplies global liver function and can be influenced by many other factors such as hyperbilirubinemia or cholestasis [4, 12, 37]. Among the eleven studies evaluating the ICG test, only five studies showed a significant difference between PHLF and non-PHLF groups. In contrast, ten out of eleven studies conducting multivariate regression analysis showed that the gadoxetic acid–enhanced MRI-derived parameters are predictive for PHLF.

The merit of using gadoxetic acid–enhanced MRI to quantitatively assess liver function is that it can potentially reduce the influence of the latent functional heterogeneity in different hepatic segments when measuring regional SI. As shown in this review, gadoxetic acid–enhanced MRI-derived parameters demonstrated such advantage in the prediction of PHLF with fairly high sensitivity (75–100%) and specificity (54–93%) in most included studies (10/15). Moreover, gadoxetic acid–enhanced MRI can also diagnose and grade liver fibrosis/cirrhosis [38, 39] with a pooled AUC of ≥ 0.92 in staging fibrosis/cirrhosis [40]. That additional information should be useful when evaluating preoperative liver function reserve. In addition, although attempts have been made to integrate ICG test or LiMAx® test and CT-based liver volumetry to improve the predictive efficiency of PHLF [41, 42], gadoxetic acid–enhanced MRI, however, can solve this problem in “one-stop shop.”

This systematic review has some limitations to be acknowledged when interpreting its results. The main limitation was the heterogeneity of the studies, not only in variance of MRI liver function parameters but also in indication and extent of the liver resection. Therefore, an initial attempt of quantitative synthesis of the results was abandoned. Future research needs to better define the inclusion criteria and adopt a more reliable gadoxetic acid–enhanced MRI parameter. Secondly, less than half of the studies (6/15) had a large sample size (more than 100 patients); only one study was prospectively designed; only two studies included both a study cohort and a validation cohort, while the others lacked internal and external validation of the efficacy of the liver function parameters. All of these potential bias sources should be fully considered when guiding future research. Thirdly, it was not possible to obtain a detailed cutoff value to recommend in clinical routine utilization from the current systematic review due to the variation among the obtained parameters in the studies. Besides, four studies in this systematic review did not supply a cutoff value for their quantitative imaging biomarker. When designing future gadoxetic acid–enhanced MRI research, it will be of great importance to use parameters that can easily be obtained also by other research groups. Fourthly, when evaluating the predictors of PHLF, half of the included studies did not take into account all important confounders (surgery-related factors were the mostly ignored). For future studies, to establish a reliable predictive model, a thorough evaluation of risk factors for PHLF is necessary. These risk factors may stem from aspects of patient (such as age, body mass index, chronic diseases), liver quality (e.g., cirrhosis, hepatitis, neo-adjuvant chemotherapy), and the surgery (such as intraoperative blood loss, time of in-flow occlusion). Lastly, attention should be paid to the reporting bias. Some included studies did not describe the process of predictive factor measurement in an explicit way. Thus, the norm of reporting gadoxetic acid–enhanced MRI research requires further standardization. Future studies need to be well-designed as prospective, multi-center trials with large sample size and utilize reliable, easily obtained liver function parameters that are independent of the MR equipment used, and they should follow a reporting norm.

To sum up, the present systematic review provides evidence that gadoxetic acid–enhanced MRI-derived parameters can serve as a promising imaging biomarker for predicting PHLF.

## Supplementary Information

Below is the link to the electronic supplementary material.

Supplementary file1 (DOC 83 KB)

## Abbreviations

AUC: Area under the receiver operating characteristic curve
DHCE-MRI: Dynamic hepatocyte-specific contrast-enhanced MRI
FLR: Future liver remnant
HUI: Hepatic uptake index
ICG: Indocyanine green
ICG-R15 test: Indocyanine green retention test at 15 min
ISGLS: The International Study Group of Liver Surgery
OATPs: Organic anion-transporting polypeptides
PHLF: Posthepatectomy liver failure
PRISMA: The Preferred Reporting Items for Systematic Reviews and Meta-Analyses guidelines
PVE: Portal vein embolization
QUIPS: The Quality In Prognostic Studies tool
RLE: Relative liver enhancement
SI: Signal intensity
