# Supplementary material for: Predictive value of gadoxetic acid–enhanced MRI for posthepatectomy liver failure: a systematic review
Source: Eur Radiol. 2021 Sep 25;32(3):1792–803. doi: 10.1007/s00330-021-08297-8 (PMC8831250; doi:10.1007/s00330-021-08297-8)
Supplement: Supplementary file 1 — Supplementary file1 (DOC 83 KB) [file 330_2021_8297_MOESM1_ESM.doc]

**Supplement file 1: literature searching strategy (as of 11^th^ December, 2020)**

Pubmed searching strategy

| #1 | Search: ((((((((("gadoxetic acid"[Title/Abstract]) OR (gadoxetate[Title/Abstract])) OR (eovist[Title/Abstract])) OR (primovist[Title/Abstract])) OR (gd-eob-dtpa[Title/Abstract])) OR ("gadolinium eob dtpa"[Title/Abstract])) OR ("gadolinium ethoxybenzyl diethylenetriamine pentaacetic acid"[Title/Abstract])) OR ("gadolinium ethoxybenzyl diethylenetriaminepentaacetic acid"[Title/Abstract])) OR ("gadolinium ethoxybenzyl dimeglumine"[Title/Abstract])) OR (gadolinium ethoxybenzyl dtpa[Supplementary Concept]) | 1,894 |
| --- | --- | --- |
| #2 | Search: (((((("liver failure"[Title/Abstract]) OR ("hepatic failure"[Title/Abstract])) OR ("liver dysfunction"[Title/Abstract])) OR (hepatic dysfunction[Title/Abstract])) OR ("liver insufficiency"[Title/Abstract])) OR ("hepatic insufficiency"[Title/Abstract])) OR ("liver failure"[MeSH Terms]) | 55,324 |
| #3 | Search: (hepatectomy[MeSH Terms]) OR (((((((hepatect*[Title/Abstract]) OR (liver surg*[Title/Abstract])) OR (hepatic surg*[Title/Abstract])) OR (liver resect*[Title/Abstract])) OR (hepatic resect*[Title/Abstract])) OR (liver segmentect*[Title/Abstract])) OR (hepatic segmentect*[Title/Abstract])) | 46,749 |
| #4 | #1 AND #2 AND #3 | 30 |

| **Supplementary table 1. Gadoxetic acid-enhanced MRI characteristics** | | | | |  |
| --- | --- | --- | --- | --- | --- |
| **Study ID** | Manufacturer | Magnetic field (T) | Dosage (mmol/kg) | Injection rate (mL/s) | HBP delay (min) |
| **Cho[21]** | GE | 1.5 | 0.025 | 2 | 20 |
| **Wibmer[22]** | Siemens | 3.0 | 0.025 | 1 | 20 |
| **Sato[23]** | GE | 3.0 | 0.1 mL/kg | N.A | 20 |
| **Jin[24]** | NA | 3.0 | 0.025 | 2 | 20 |
| **Costa[25]** | Siemens | 1.5, 3.0 | 0.025 | N.A | 20 |
| **Asenbaum[26]** | Siemens | 3.0 | 0.025 | 1 | 20 |
| **Chuang[27]** | GE | 1.5 | 0.025 | 1 | 30 |
| **Kim[28]** | Siemens | 3.0 | 0.1 mL/kg | 1 | 20 |
| **Theilig[29]** | Siemens | 1.5 | 0.1 mL/kg | N.A | 20 |
| **Araki[30]** | Siemens | 3.0 | 0.1 mL/kg | N.A | 20 |
| **Donadon[31]** | Siemens | 1.5 | 0.025 | N.A | 20 |
| **Orimo[32]** | Philips | 1.5 | 0.1 mL/kg | 2 | 20 |
| **Zhu[33]** | Philips | 3.0 | 0.025 | 2.5 | 20 |
| **Tsujita[34]** | Philips | 3.0 | 0.1 mL/kg | 1-2 | 20 |
| **Wang[35]** | Siemens | 3.0 | 0.025 | 2 | 15 |

Note: HBP, hepatobiliary phase; N.A, not available.

Studies excluded in eligibility assessment

During eligibility assessment of the 22 studies with full-text were obtained for further. The detailed excluded reasons are listed as follows:

Repeated study (n=1): [1]

MRI used for liver volumetry (n=1): [2]

PHLF involved both PVE and liver resection (n=1):[3]

= 4me not related to PHLF ()er resction term to summary the indications in Cho and Chuang'00000000000000000000000000000000000

Outcome not related to PHLF (n = 4): [4-7]

1 Costa AF, St-Germain AT, Abdolell M, Smoot RL, Cleary S, Jhaveri KS (2018) How Do Different Indices of Hepatic Enhancement With Gadoxetic Acid Compare in Predicting Liver Failure and Other Major Complications After Hepatectomy? J Comput Assist Tomogr 42:380-386. https://doi.org/10.1097/rct.0000000000000691

2 Yamada S, Shimada M, Morine Y, et al (2019) A new formula to calculate the resection limit in hepatectomy based on Gd-EOB-DTPA-enhanced magnetic resonance imaging. PLoS One 14https://doi.org/10.1371/journal.pone.0210579

3 Barth BK, Fischer MA, Kambakamba P, Lesurtel M, Reiner CS (2016) Liver-fat and liver-function indices derived from Gd-EOB-DTPA-enhanced liver MRI for prediction of future liver remnant growth after portal vein occlusion. Eur J Radiol 85:843-849. https://doi.org/10.1016/j.ejrad.2016.02.008

4 Geisel D, Ludemann L, Keuchel T, et al (2013) Increase in left liver lobe function after preoperative right portal vein embolisation assessed with gadolinium-EOB-DTPA MRI. Eur Radiol 23:2555-2560. https://doi.org/10.1007/s00330-013-2859-1

5 Yoon JH, Choi JI, Jeong YY, et al (2016) Pre-treatment estimation of future remnant liver function using gadoxetic acid MRI in patients with HCC. Journal of hepatology 65:1155-1162. https://doi.org/10.1016/j.jhep.2016.07.024

6 Araki K, Harimoto N, Yamanaka T, et al (2020) Efficiency of regional functional liver volume assessment using Gd-EOB-DTPA-enhanced magnetic resonance imaging for hepatocellular carcinoma with portal vein tumor thrombus. Surg Today. 10.1007/s00595-020-02062-yhttps://doi.org/10.1007/s00595-020-02062-y

7 Itoh S, Yoshizumi T, Shirabe K, et al (2017) Functional remnant liver assessment predicts liver-related morbidity after hepatic resection in patients with hepatocellular carcinoma. Hepatology research : the official journal of the Japan Society of Hepatology 47:398-404. https://doi.org/10.1111/hepr.12761

**Embase searching str**ategy

| #1 | 'gadoxetic acid'/exp OR 'gadoxetic acid' OR eovist:ab,ti OR 'gadolinium eob dtpa':ab,ti OR 'gadolinium ethoxybenzyl diethylenetriamine pentaacetic acid':ab,ti OR 'gadolinium ethoxybenzyl diethylenetriaminepentaacetic acid':ab,ti OR 'gadolinium ethoxybenzyl dtpa':ab,ti OR gadoxetate:ab,ti OR primovist:ab,ti OR 'gd-eob-dtpa':ab,ti OR 'gadolinium ethoxybenzyl dimeglumine':ab,ti | 3,440 |
| --- | --- | --- |
| #2 | 'liver failure'/exp OR 'liver failure' | 87,729 |
| #3 | 'liver dysfunction':ab,ti OR 'liver insufficiency':ab,ti OR 'hepatic failure':ab,ti OR 'hepatic dysfunction':ab,ti OR 'hepatic insufficiency':ab,ti | 33,302 |
| #4 | #2 OR #3 | 106,880 |
| #5 | 'hepatectomy'/exp OR hepatectomy | 64,572 |
| #6 | 'liver surg*':ab,ti OR 'hepatic surg*' OR 'liver segmentect*':ab,ti OR 'hepatic segmentect*':ab,ti OR 'liver resect*':ab,ti OR 'hepatic resect*':ab,ti OR 'hepatect*':ab,ti | 55,137 |
| #7 | #5 OR #6 | 72,300 |
| #8 | #1 AND #4 AND #7 | 49 |

**W**eb of Science searching strategy

| #1 | TS=(gadoxetate OR "Gd-EOB-DTPA" OR "gadolinium ethoxybenzyl diethylenetriaminepentaacetic acid" OR "gadolinium ethoxybenzyl diethylenetriamine pentaacetic acid" OR "gadoxetic acid" OR "gadolinium eob dtpa" OR Eovist OR Primovist OR "gadolinium ethoxybenzyl DTPA" OR "gadolinium ethoxybenzyl dimeglumine") | 2,160 |
| --- | --- | --- |
| #2 | TS=("liver failure" OR "hepatic failure" OR "liver dysfunction" OR "hepatic dysfunction" OR "liver insufficiency" OR "hepatic insufficiency") | 40,312 |
| #3 | TS=("liver surg*" OR "hepatic surg*" OR "liver resect*" OR "hepatic resect*" OR "liver segmentect*" OR "hepatic segmentect*" OR "hepatect*") | 40,039 |
| #4 | #1 AND #2 AND #3 AND LANGUAGE: (English) | 34 |

Cochrane searching strategy

| #1 | ("gadoxetic acid"):ti,ab,kw OR ("Gd-EOB-DTPA"):ti,ab,kw OR ("gadolinium ethoxybenzyl diethylenetriaminepentaacetic  acid"):ti,ab,kw OR ("gadolinium ethoxybenzyl dimeglumine"):ti,ab,kw OR ("gadolinium ethoxybenzyl diethylenetriamine pentaacetic acid"):ti,ab,kw OR (gadoxetate):ti,ab,kw OR (Eovist):ti,ab,kw OR (Primovist):ti,ab,kw OR ("gadolinium ethoxybenzyl DTPA"):ti,ab,kw OR ("gadolinium eob dtpa"):ti,ab,kw | 70 |
| --- | --- | --- |
| #2 | MeSH descriptor: [liver failure] explode all trees | 859 |
| #3 | ("liver failure"):ti,ab,kw OR ("liver dysfunction"):ti,ab,kw OR ("liver insufficiency"):ti,ab,kw OR ("hepatic failure"):ti,ab,kw OR ("hepatic dysfunction"):ti,ab,kw OR ("hepatic insufficiency"):ti,ab,kw | 4,069 |
| #4 | #2 OR #3 | 4,546 |
| #5 | MeSH descriptor: [Hepatectomy] explode all trees | 614 |
| #6 | (liver surg*):ti,ab,kw OR (hepatic surg*):ti,ab,kw OR (liver resect*:ti,ab,kw) OR (hepatic resect*:ti,ab,kw) OR (liver segmentect*:ti,ab,kw) OR (hepatic segmentect*:ti,ab,kw) OR hepatect*:ti,ab,kw | 11,689 |
| #7 | #5 OR #6 | 11,689 |
| #8 | #1 AND #4 AND #7 | 1 |
